# Facilitating children’s communication in problem-solving activities with a coding toy: teachers’ semiotic mediation in early childhood education and care

**DOI:** 10.3389/fpsyg.2024.1426165

**Published:** 2025-01-10

**Authors:** Francesca Granone, Enrico Pollarolo

**Affiliations:** Department of Early Childhood Education, University of Stavanger, Stavanger, Norway

**Keywords:** problem-solving, early childhood education, teachers, coding toys, communication, semiotic mediation

## Abstract

This study investigates the role of teacher mediation in facilitating children’s communication during problem-solving, play-based coding activities with Kubo, a screen-free coding toy, in Early Childhood Education and Care (ECEC) settings. Following an initial observation involving nine kindergarten teachers and 36 children, a workshop was held to identify elements that teachers considered relevant for facilitating children’s use of verbal and non-verbal communication. Key mediation elements, such as multimodal communication, planning, time, humor, and reflective questioning, were identified during the workshop and applied in a subsequent observation with the same participants. The findings reveal that these mediation strategies facilitated children’s communication and participation in the activity using a multimodal approach to support their problem-solving process. Teacher mediation facilitated children’s ability to articulate their thought processes, fostering a communicative and reflective learning environment. This study underscores the importance of various elements in teachers’ semiotic mediation and identifies specific strategies that show promise for engaging all children.

## 1 Introduction

Developing children’s problem-solving skills is one of the primary goals of education (Keen, 2011, 398; Voss, 2012). Several studies have explored the importance of promoting problem-solving skills in school children and higher education students (Fawcett and Garton, 2005; Beyazsacli, 2016; Ahghar, 2012; Saputro et al., 2019). Research on problem-solving skills in early childhood is often linked with mathematics (Lossius and Lundhaug, 2020; Meaney et al., 2016; Lopes et al., 2017; Pollarolo et al., 2023; Reikerås et al., 2012), with a focus on teachers’ approach to problem-solving (Fosse et al., 2020). Further, broad consensus has been established about the importance of fostering problem-solving skills at the Early Childhood Education and Care (ECEC) level (Hollenstein et al., 2022; Lopes et al., 2017; Whittaker and McMullen, 2014; Reikerås et al., 2012).

Play-based activities in early childhood education are crucial for children’s development. Introducing digital toys in ECEC, particularly within play-based activities, may foster problem-solving abilities along with other essential skills (Çiftci and Bildiren, 2020; Heikkilä and Mannila, 2018; Liu et al., 2013; Nam et al., 2019; Otterborn et al., 2020; Shumway et al., 2019; Granone and Reikerås, 2021; Undheim, 2022). In these types of play-based activities, the role of the teacher is always essential because the tool alone is not effective for children’s learning (Quintana et al., 2018; Reiser, 2018; Kozulin, 2002). If the interaction between the toys and the children is mediated, the artefact can be appropriated by the children (Kozulin, 2002; Granone and Reikerås, 2023). This mediation can be done through verbal, graphical-written forms or through gestures (Bartolini Bussi and Baccaglini-Frank, 2015). Effective communication in early childhood is often multimodal, as young children rely on gestures alongside speech to express their ideas and solve problem-solving tasks. Research highlights that gestures are an integral element to young children’s expression of thoughts, enabling them to communicate aspects of understanding that may not yet be accessible through verbal language alone (Johansson and Johansson 2014). Children use gestures to express intentions, explore possibilities, and clarify their thinking, revealing insights into their cognitive processes that might otherwise remain unspoken (Johansson and Johansson 2014). Teachers play a crucial role in supporting these multimodal expressions. Literature underscores that gestures, when encouraged and interpreted by teachers, can deepen children’s engagement with problem-solving activities and facilitate their expression of reasoning (Johansson and Johansson 2014). In problem-solving tasks, gestures are essential tools that help children externalize their thoughts, organize information, and communicate ideas that may be challenging to verbalize for pre-school children. When children point, trace, or mimic actions, they are not only guiding their own focus but also structuring their thought processes in ways that make problem-solving more accessible and collaborative. These gestures allow children to visualize sequences, test out solutions, and express their reasoning before committing to specific actions (Goldin-Meadow, 2005). Research indicates that gestures enhance spatial and sequential thinking, enabling children to break down complex tasks into manageable steps and simulate outcomes in a tangible way, which is particularly valuable in early childhood settings where abstract verbal expression may still be developing (Broaders et al., 2007; McNeill, 2019). Recognizing and supporting gestures in problem-solving activities encourages children to express and refine their ideas, fostering a multimodal approach to learning.

Literature shows that in many ECEC settings, verbal guidance tends to dominate teacher support (Pollarolo et al., 2024), with limited emphasis on acknowledging or encouraging non-verbal communication. This suggests the need for developing more research to support teachers in integrating both verbal and non-verbal forms of communication for children’s investigation of a play-based activity with a coding toy focused on problem-solving. Given the key role of teachers in this mediation, this study actively involves the teachers themselves in the research process to capture their perspectives and interpretations. This approach allows for gathering deeper insights into teachers’ views on mediating verbal and non-verbal forms of communication to support children’s exploration and problem-solving in play-based activities.

The research question identified for the present study is “What mediation elements can teachers identify as relevant to facilitate children’s communication (verbal and non-verbal) during problem-solving play-based activities with a coding toy?”

In line with the Norwegian ECEC perspective (Kunnskapsdepartementet, 2017), the focus of this study is on facilitating children’s exploration and supporting their problem-solving process rather than measuring predefined learning outcomes. The teachers’ role emphasizes creating an environment where children are encouraged to experiment, reflect, and develop their reasoning skills, guided by thoughtful mediation. This process-oriented approach prioritizes fostering curiosity, communication, and engagement as integral to holistic development, differing from outcome-based approaches that assess learning primarily through quantifiable results. By focusing on the process, the study highlights the importance of facilitating children’s active communication and participation and growth in a collaborative learning environment. This study contributes to the integration of technology in early childhood education by highlighting how coding toys can be used in play-based learning activities to facilitate communication. By focusing on teacher mediation strategies within the Norwegian ECEC context, the research underscores the importance of multimodal approaches in supporting children’s holistic development.

## 2 Background

### 2.1 Problem-solving: Polya’s four phases

Norwegian Early Childhood Education and Care settings emphasize the importance of children’s ability to tackle challenges (Kunnskapsdepartementet, 2017). Problem-solving is a multifaceted concept with numerous interpretations (Granone et al., 2023; Liljedahl and Cai, 2021). For instance, the literature has explored problem-solving from various perspectives, characterizing it as a cognitive procedure aimed at overcoming obstacles or as a combination of cognitive functions like attention, memory, language, and metacognition (Simamora and Saragih, 2019; Yayuk and Husamah, 2020; Güner and Erbay, 2021). As an educational objective, problem-solving is viewed as a skill children can develop through diverse methods, including technology (Granone and Reikerås, 2021) or outdoor experiences (Lossius and Lundhaug, 2020). However, problem-solving can also serve as a teaching approach, particularly in subjects such as mathematics (Brijlall, 2015). Polya (2004) delineated problem-solving into four stages: understanding the problem, making a plan, executing the plan, and looking back. Each stage contributes to a deeper comprehension of both the problem itself and the problem-solving process (Polya, 1971).

Polya (1971) articulated that the problem-solving process encompasses several distinct phases. Initially, in the understanding the problem phase, the problem solver examines the problem statement, identifying pertinent information and constraints. Additionally, he/she seeks clarification through questioning, delineating the problem’s requirements and discerning the knowns and unknowns. Drawing upon relevant concepts and methodologies, a plan is then formulated in the make a plan phase. Here, the solver may break down the problem into more manageable components and explore potential strategies and tools for resolution. The formulated plan is then executed in the carry out the plan phase. Each step is methodically implemented, employing mathematical or logical reasoning as necessary. Finally, in the looking back phase, the solution is critically reviewed for accuracy and coherence within the problem’s context. The solver reflects on the problem-solving process, extracting insights that may inform future endeavors. Furthermore, consideration is given to alternative solutions or more efficient methods.

Of these stages, the first phase—understanding the problem—is particularly crucial, as it enables the problem solver to clearly identify what needs to be comprehended and resolved (Polya, 1971). This foundational understanding is essential for guiding the subsequent phases of planning, executing, and evaluating, as it provides the clarity and direction necessary to move effectively through the entire problem-solving process (Polya, 1971; Granone et al., 2023).

### 2.2 Coding toys and teachers’ mediation

Digital technology can influence teaching and learning from early childhood education and care (Donohue, 2014; Keengwe and Onchwari, 2009; Bourbour, 2023) to higher education (Lai, 2011). In particular, coding toys foster children’s coding abilities, which positively affect other key skills, such as problem-solving and analytical reasoning (Çiftci and Bildiren, 2020; Granone and Reikerås, 2021). For example, through interactions with a tangible user interface, children can develop the ability to transfer cognitive abstracts into physical behaviors (Lin et al., 2020). Kubo’s physical components, such as the tactile tiles with directional arrows, serve as tangible tools that facilitate children’s understanding of sequencing and directionality. By physically manipulating the tiles to create a path for Kubo, children engage in hands-on exploration of problem-solving concepts. The arrangement of tiles requires children to plan, predict outcomes, and adjust their strategies based on immediate feedback from the robot’s movements. This tactile interaction bridges the gap between abstract concepts and concrete experiences, fostering a problem-solving mindset.

The role of teacher mediation has been explained in the theory of semiotic mediation (Bartolini Bussi and Baccaglini-Frank, 2015), where teachers play a dual role in planning and mediating the activities to support learning. In the planning phase, teachers carefully select tasks, artefacts, and goals that align with specific mathematical concepts. This stage is essential for identifying the semiotic potential of coding toys and how they can best promote desired learning outcomes (Bartolini Bussi and Mariotti, 2008). During mediation, teachers facilitate children’s engagement with tasks by providing interactive support, encouraging them to observe, reflect, and communicate their ideas about actions and choices. Research highlights that gestures are an integral part of children’s multimodal communication, helping them expressing complex thoughts that may not yet be fully accessible verbally (Goldin-Meadow, 2003; McNeill, 1992). Children’s gestures, such as pointing or mimicking a robot’s movement, provide insight into their problem-solving process and often reveal implicit understanding that aids in learning (Broaders et al., 2007). Teachers can support these gestures to bridge children’s immediate, situated understanding (e.g., using tiles to indicate direction) with more abstract mathematical concepts like sequencing and spatial reasoning (Roth, 2001). They guide children in producing and interpreting signs associated with both the artefact (situated signs) and mathematical concepts (mathematical signs). Situated signs, like the physical tiles used to code the robot’s path, allow children to understand the robot’s movement in the immediate context. In contrast, mathematical signs, such as sequencing steps or recognizing patterns in movement, bridge this situated understanding to broader mathematical concepts, like spatial reasoning and problem-solving. Through mediation, teachers help children transition from using artefact-specific signs to expressing mathematical ideas, fostering deeper understanding and supporting children’s ability to communicate their reasoning (Bartolini Bussi and Mariotti, 2008; Alibali and Nathan, 2012).

### 2.3 The present study

The coding toy used in this study is a robot called Kubo that can be programmed without screens by puzzling different tactile tiles with arrows drawn on them (Bertel et al., 2019). As an artefact, Kubo provides stimuli that teachers can leverage to scaffold children’s problem-solving skills (Bartolini Bussi and Mariotti, 2008). In this context, artefact signs include the directional arrows used to build a path for Kubo, while mathematical signs are associated with concepts like orientation and counting, both useful in the chosen problem-solving play-based activity. Two pivotal problem-solving signs emerge in these activities: the individual arrows, representing specific steps in the solution, and the overall path, representing the strategy needed to achieve the goal. Together, these signs guide children through the process of breaking down, sequencing, and then solving the play-based activity presented.

In our study, the teachers designed an activity specifically to guide children in understanding a problem-solving task. This article focuses on data from the observations related to the initial phase of Polya’s problem-solving model (1974), in order to have data from Observation 0 and Observation 1 that could be more comparable. In this phase the teachers could support children in breaking down the problem, recognizing important information, and connecting prior knowledge to the task at hand. Polya’s problem-solving description was selected among others because the teachers involved in the project had already familiarity with this model. This familiarity among the teachers provided a common framework for analyzing problem-solving in play-based coding activities. Its emphasis on understanding the problem as the foundational step closely aligns with the goals of the study, as this phase is essential for supporting young children in articulating, exploring, and breaking down tasks. By using a model known to teachers, we aimed to ensure consistency in mediation approaches and to facilitate teacher engagement with the problem-solving process in ways that were accessible.

## 3 Methods

### 3.1 Participants

The present study is part of a research project named “DiCoTe.” DiCoTe stands for “Enhancing professional Digital Competence in Early Childhood Teacher Education with a focus on enriching and supporting children’s play with coding toys.” The project aims to contribute to the development of research expertise that addresses significant societal challenges by defining activities that enrich and support children’s play with technology in ECEC settings.

Nine ECEC teachers, eight women and one man aged between 31 and 56 years, from two different municipalities participated in the present study, with 36 4-year-old children. While the children included in the overall project ranged in age from 3 to 5 years old, the children participating in the present study were between 4 years and 1 month and 4 years and 9 months old. The teachers had extensive experience as pedagogical leaders, ranging from 10 to 30 years, which enabled them to tailor the activities to suit the children’s ages. While many of them had prior experience with technology, two had limited experience but demonstrated some familiarity. None of the teachers had worked with Kubo before this study. Each teacher developed a play-based activity (improvised in Observation 0, planned in Observation 1) with the coding toy Kubo for a group of four children. Both municipalities involved were interested in increasing teachers’ competence in technology, particularly regarding coding toys.

Conducting research with children demands special ethical considerations (Fine and Sandstrom, 1988). Informed consent was obtained from the teachers and the parents. The teachers were already familiar with the children and were therefore responsible for explaining the study to them in a way they could understand. The children were informed that they could indicate verbally or through other means if they did not want to be observed at any time during the project. The children’s names have been anonymized to comply with the ethical guidelines of the Norwegian Agency for Shared Services in Education and Research, which authorized the project. The data were stored in a secure server, and only the project leader and the researchers involved had access to this information.

### 3.2 Procedure

This study was conducted in several stages: Observation 0 (observation of the play-based activities in each ECEC institution), a workshop where the teachers could discuss and reflect on what happened in Observation 0 and identify important mediation elements that could be relevant for facilitating children’s communication during a problem-solving play-based activity with Kubo, Observation 1 where the teachers could prepare problem-solving play-based activity with Kubo taking into consideration the mediation elements identified during the workshop and shared in a common online platform (Microsoft Teams). The mediation elements were identified for facilitating children’s communication, exploration and engagement, as for example gestures for facilitating children in directing their attention and establishing a shared focus, or hand movements that mirrored the robot’s actions to guide and support children’s collaborative engagement in the problem-solving process. More detailed descriptions of the mediation elements, particularly related to communication, are reported in the results of this study. Although the present study focuses on mediation elements particularly related to communication, these have also influenced exploration and engagement.

Between Observation 0 and the workshop, and from the workshop to Observation 1, the teachers worked with the children regularly, using the robot once a week to explore various activities they had designed themselves and shared on the platform in Teams. After each session, they documented their observations in a log, which they then shared with the first author for further analysis. Data from Observation 0 served as the foundation for developing the workshop, where teachers could reflect collaboratively and identify what they considered useful mediational elements for facilitating children’s communication during problem-solving, play-based activities with a coding toy. These identified mediational elements were then implemented regularly with children in subsequent sessions and were central to Observation 1. The goal of Observation 1 was not to measure children’s competence, but rather to observe whether teachers had gained confidence in facilitating children’s communication and engagement during problem-solving activities.

#### 3.2.1 Observation 0

Observation 0 was conducted in June 2022. Kubo was presented to the ECEC teachers and the children for the first time to observe their interactions with Kubo. Teachers were not given instructions on how to introduce or conduct the activities involving Kubo with the children, neither on how to support children’s communication. None of the children had previous experience with Kubo. The children and their teachers were video-recorded while they played with Kubo. The activities were performed in each ECEC institution in approximately half an hour.

In each ECEC institution, the activities were conducted in a dedicated room to allow the children to work in a familiar but quiet environment. The video camera was fixed in position to capture the children’s dialogue, language, and gestures. This approach ensured the collection of rich data necessary for multimodal analysis. To enable the teachers to focus entirely on interacting with the children, they were asked not to handle the camera or take any notes during the session. The video-recorded activities (a total of 444 min) were transcribed and controlled by more than one researcher.

#### 3.2.2 Workshop

In September 2022 a one-day workshop was held with all the teachers involved in the study. The focus of the workshop was to help the teachers reflect on Observation 0 and children’s communication, exploration, and engagement. In the first part of the workshop, the researchers presented reflections and posed questions to the teachers related to Observation 0. The teachers were then divided into groups and had the opportunity to work with the Kubo robot again, reflecting collaboratively on their experiences during Observation 0 and the regular weekly activities they had conducted with the children. During the workshop, which was part of the planned activities within the long-term project DiCoTe, of which the present study is a part, the teachers documented their reflections and notes about the activities they had carried out so far and the tasks completed during the workshop. They highlighted elements they identified as relevant for facilitating children’s communication, exploration, and engagement. These notes were later transcribed for analysis through thematic analysis, and the mediation elements identified by the researchers and teachers, which form part of the results presented in this study, were shared on a common Teams platform. This platform was intended to inspire teachers in defining activities and mediating children’s communication, exploration, and engagement during the activities they regularly developed with the children and particularly during Observation 1. Although the study explored various abilities, children’s communication was a key focus. Building on the insights gained from Observation 0 and the workshop, the aim was for teachers to plan a structured, play-based activity where they could support children’s multimodal communication. By focusing on multimodal support, including both verbal and non-verbal cues, and creating problem-solving scenarios, teachers could better guide children’s exploration and collaborative interactions.

#### 3.2.3 Observation 1

Six months after the workshop, in December 2022, the teachers engaged in additional activities with the same coding toys, involving, where possible, the same children. During this phase, a total of 358 min of video data were recorded and transcribed.

### 3.3 Analysis

The video data were observed, analyzed, and transcribed. Subsequently, the researchers discussed the data to reach agreement regarding the results. A content analysis of the video-recorded play was conducted. Content analysis is defined as “a research technique for making replicable and valid inferences from texts (or other meaningful matter) to the contexts of their use” (Krippendorff, 2018, p. 24). Content analysis helped identify the interactions that occurred between teachers and children and the type of mediation provided by the teachers (non-verbal or verbal). The most representative data are reported in the tables.

Thematic analysis was conducted on the transcriptions of the notes from the workshop. The analysis began with familiarization, during which each researcher took notes through multiple readings of the data. This was followed by data coding using an inductive approach. While the approach was inductive, it is important to acknowledge that a purely inductive process is not entirely possible, as researchers inevitably bring their own perspectives to the data analysis to some extent (Braun and Clarke, 2006).

To ensure the reliability and validity of the content and thematic analyses, several steps were taken. The two authors independently reviewed and coded the video transcripts and workshop notes. Inter-coder reliability was assessed by comparing codes and resolving discrepancies through discussion until consensus was reached. Triangulation was employed by cross-referencing data from observations, teacher notes, and video recordings. Additionally, member checking was conducted by sharing preliminary findings with the participating teachers for their feedback and validation.

## 4 Results

### 4.1 Results from Observation 0

The following table reports the data collected during Observation 0. It summarizes the activities improvised by the teacher (situation during the activity), children’s gestures, children’s speech, teachers’ mediation (non-verbal), teachers’ mediation (verbal), and children’s reaction to teachers’ mediation. As shown in Table 1, the teachers were not consistently able to respond to suggestions from all the children, focusing primarily on verbal communication while giving less attention to non-verbal cues. This imbalance appeared to impact the children’s engagement, which seemed to decrease following interactions with the teacher.

**Table 1:** Observation 0.

Teacher	Situation during the activity	Children’s gestures	Children’s speech	Teachers’ reaction (gestures)	Teachers’ reaction (speech)	Children’s reaction
1	Children and the teacher are sitting on the floor. They started to program, and C1 is trying to decide where the Kubo robot should go next.	He points to the left arrow tile, signaling that Kubo should turn left.	C1: “Kubo should go there!”		Teacher: “Let us move forward now.”	C1 stops pointing and just says, ‘Okay, forward.’
	C2 is unsure which tile to place next to guide Kubo around an obstacle.	She waves her hand over the tiles, showing indecision.	C2: “‘I think it should go around here.”		Teacher: “Place an arrow to go straight ahead.”	C2 stops waving her hand and puts down a straight arrow without further comment.
2	The children are building a path, the teacher observes. The teacher ask a question “If we want to go from the school to the bus, what should we do?”	C3 points to the school. He slowly moves the finger from the school position to the bus, going through the road on the left of the playground.	C4 “We go to the candy shop before!”		Teacher: “C4, you want to try this? Okay.”	C4 starts to build the path, while C3 looks away.
	The children have built part of the path, and now they need to turn left. The children seem stuck.				Teacher: “What happen if you put an orange arrow after that? Can you say it, before doing it?”	Each child moves the hands, trying to understand the result of putting an orange arrow. No one talks. The teacher put the arrow and ask the children to go on.
3	The children are sitting on the floor with the teacher. C5 has the robot in the hands, and the arrows are on the floor near the cardboard.				Teacher: “Where do you want to start? Who want to tell me?”	Children has no experience with the robot and are not able to understand how to start. They seem not to feel free to take action.
	Children have finished a path from the bus to the playground. Now they want to build a new one.	C6 point to the fire. C7 nods.			Teacher: “Can we start from the bus, but this time to move to the candy shop?”	C5 starts building the new path.
4	The children investigate the robot and the different element. The teacher investigates alone without interacting with the children.					
5	The children are sitting on the floor with the teacher. C8 has the robot in the hands, and the arrows are on the floor near the cardboard.				Teacher: “Where do you want to go?”	Children has no experience with the robot and are not able to understand how to start. They seem not to feel free to take action.
	The children have built part of the path. C9 think that the path has a mistake.	C9 point to a blue arrow in the path.	C9: “It’s wrong!”		Teacher: “Where do you want to go?”	C9 stop pointing. All the children think about what could be wrong.
6	The children have placed several arrows on the path but are unsure about how to make Kubo turn at the corner.	C10 points to a right arrow while moving their hand in a turning motion.	C1: “Maybe we should make Kubo turn here?”		Teacher: “That looks like a good idea! Let us try turning Kubo to the right.”	C10 smiles and places the right arrow in the path.
	The children are halfway through the path, but C11 is unsure if they should go straight or turn.	C11 taps the straight arrow repeatedly but glances at the left arrow.	C12: “We should go left”		Teacher: “I understand that there are different opinions here. C12 has an opinion. Do you agree C11?”	C12 puts the arrows in the path, C11 think.
7	The children have finished a path and want to test it. C13 notices something might be wrong.	C13 points to a corner in the path.	C13: “I think we should have turned earlier.”		Teacher: “You might be right! Let us go back and see where we could have turned.”	The children look at the path together, the teacher points to the starting point and move the finger along the path until it reaches the destination.
	The children are starting a new path, but C14 is unsure how to begin.	C14 takes a green arrow, but then she stops. C15 takes a blue arrow and place it in the position where a bus id designed.	C15: “I start”		Teacher: “I see that C14 would like to place an arrow too. Is it ok for you to place it now, or you wanted to put it in the starting point?”	C15 stops, and all the children wait. C14 smiles putting the green arrow after the blue one.
8	The children are sitting on the floor with the teacher. C16 has the robot in the hands, and the arrows are on the floor near the cardboard.				Teacher: “Where do you want to go? To the school? To the bus?”	
	C17 seems unsure in the activity and is not participating with the other children.	C17 observes the activity but is never interacting with the other children or touching the tactiles.			Teacher: “Now is C17 time. Please choose where you want to go. Forward? Left? Right? We know that the green arrow follows a forward path. Do you agree to use it?”	The other children wait. The teacher gives a green arrow to C17. C17 thinks for a while, then he places the green arrow to continue the path.
9	The children investigate the robot and the different element. The teacher investigates alone without interacting with the children.					

### 4.2 Results from the workshop

The data from the workshop reveal several elements that teachers identified as essential for facilitating children’s communication when using coding toys. Teachers emphasized the importance of using verbal guidance to encourage children to think through their actions, often asking questions such as, “What do you think will happen?” or “Can you describe your actions?”. This approach aligns with the belief that communication extends beyond instructions, as teachers actively asked children to describe their actions to help them reflect on their choices.

Teachers also observed that body language plays a crucial role in facilitating children’s understanding. Gestures, such as pointing at tiles, helped children direct their attention and establish a shared focus. Teachers used hand movements that mirrored the robot’s actions to guide and support children’s collaborative engagement in the problem-solving process. In addition to body language, teachers highlighted visual aids and physical interaction as essential components of their approach. One teacher noted, “Physical guidance is better than verbal guidance,” while another remarked, “Demonstrating movements helps children understand how to place the tiles and predict Kubo’s path.” Teachers recognized that showing how Kubo moves on the tiles provided immediate feedback, enabling children to learn through observation.

The teachers’ notes also emphasized the importance of facilitating children throughout the investigation process, encouraging independent thinking. For example, teachers expressed their thoughts by stating, “It is important to let the children think for themselves” and “Follow the children’s lead, letting them figure it out.” However, they acknowledged that some children needed additional physical and visual scaffolding. One teacher commented, “We showed how the arrows work by pointing to the direction and demonstrating the movement.”

Teachers also reflected on the role of visual guidance in supporting children’s understanding. One teacher noted, “Describing the pictures on the cardboard could help children understand both spatial and coding concepts in ways that verbal instructions alone cannot.” Another teacher highlighted the importance of engaging all children by allowing sufficient time for responses, observing, “Quick responses may reduce children’s motivation to communicate their thoughts.” Furthermore, humor was identified as a tool for reducing stress in situations where children seemed confused.

The data underscore the importance of planning, with teachers stressing the need for structured activities to support children’s exploration and communication. Two teachers reflected, “Receiving a coding toy without prior knowledge and trying it out with children was challenging.” Another teacher commented, “Planning helps guide children toward more complex tasks,” emphasizing the importance of understanding the problem in advance and explaining it step-by-step. At the same time, flexibility emerged as a recurring theme. Teachers noted that following the children’s initiatives allowed for richer, more exploratory learning experiences. They stated, “We should plan specific goals but leave room for children’s exploration” and “A defined goal helps, but children should also have the freedom to explore.” Teachers emphasized the need for a balance, where structured goals guide the activity but still allow space for children to freely plan, solve problems, and communicate their ideas.

### 4.3 Results from Observation 1

The following table reports the data collected during Observation 1. It includes details about the activity planned by the teacher (situation during the activity), children’s gestures, children’s speech, teachers’ mediation (gestures), teachers’ mediation (speech), and children’s reaction to teachers’ mediation. As illustrated in Table 2, the teachers displayed greater engagement and attentiveness to all forms of communication, both verbal and non-verbal. This increased awareness allowed teachers to recognize when a child’s verbal expression differed from their non-verbal cues, facilitating more inclusive participation and enhancing the children’s overall communication during the activity.

**Table 2:** Observation 1.

Teacher	Situation during the activity	Children’s gestures	Children’s speech	Teachers’ reaction (gestures)	Teachers’ reaction (speech)	Children’s reaction
1	The children and the teacher are building a path for programming the robot.	C1 points toward the robot’s path, indicating his plan to move the robot.		The teacher acknowledges the gesture by nodding	The teacher acknowledges the gesture asking, ‘Are you showing me where the robot should go next?’	The child continues using gestures to communicate his strategy.
	The children are setting up the path for Kubo, but they hesitate after placing the first arrow.	C1 points to the next arrow (a forward arrow) near the cardboard but does not place it yet.	C1: “Should it go straight or turn?”	The teacher nods and points toward the forward arrow.	“What do you think will happen if Kubo goes straight?”	C1 places the forward arrow, saying, “It will keep going, I think.”
2	The children face an obstacle in Kubo’s path and need to decide how to navigate around it.	C2 waves their hand over the tiles, showing indecision about the next step.	C2: “It could go here or here.”	The teacher traces the possible path options with their finger.	“Let us think: if we go this way, where will Kubo end up?”	C2 picks the left arrow and says, “It will go around this way.”
	The children are choosing between two arrows, unsure which direction will lead Kubo to the goal.	C3 holds both a right and a forward arrow, looking between them.	C3: “Should it turn or go forward?”	The teacher points to the forward arrow.	“Remember last time? What did we do when we wanted to go straight?”	C3 nods and places the forward arrow, saying, “We went straight, just like now.”
3	The children have almost completed their path but need one final arrow to reach the goal.	: C4 points to the end point but hesitates before placing the last arrow.	“Will this make Kubo reach the end?”	The teacher waits and give C4 the time for thinking. She sees that C4 tries to trace the path with the finger, and then traces the path with her finger leading C4’s hand to visualize the final move.	“Take your time C4, just think. What happens if you place that arrow? Where will Kubo go?”	C4 places the arrow and says, “It will reach the end now.”
	The children are uncertain about how to handle a tricky turn in the path.	C5 moves their hand in a turning motion but does not place the arrow.	C5: “Will Kubo turn here?”	The teacher mirrors the turning gesture with their hand.	“What do you think will happen if we use the turn arrow?”	C5 smiles and places the arrow, saying, “It will turn just like this!”
4	The children are building a new path but are stuck on how to start it.	C6 points to the starting tile and looks at the teacher.	C6: “Should we start with this one?”	The teacher nods and points to the first arrow.	“Yes, let us think. What will Kubo do if you start here?”	C6 places the first arrow, saying, “It will go straight to the next tile.”
	The children notice that part of their path is not working as expected.	C7 points to an arrow that seems out of place.	C7: “I think this one is wrong.”	The teacher points to the beginning of the path and traces the sequence.	“Let us go back and see where we can fix it.”	C7 nods and replaces the arrow, saying, “Now it will work!”
5	The children need to adjust their path but are unsure how to proceed.	C8 holds a turning arrow and moves it back and forth.	C8: “Should it go here or there?”	The teacher gestures toward the path and points to where the turn should go.	“What happens if you put the turn here?”	C8 places the arrow, saying, “It will go around the corner now.”
	The children are completing a complex path and need to make sure Kubo reaches the end	C9 points to the end goal and the final arrow.	C9: “Will this be the last one?”	The teacher traces the final section of the path with their finger.	“Yes, this is the last step. What will happen if you place this arrow?”	C9 smiles and places the arrow, saying, “It will finish the path!”
6	The children have successfully navigated part of the path but face a new challenge with a sharp turn.	C10 points to the turning arrow, then looks at the teacher for confirmation.	C10: “Is this the right one for turning here?”	The teacher nods and mimics a turning motion with their hand.	“Yes, if you place the turn here, where will Kubo go next?”	C10 places the turning arrow and says, “It will go around the corner!”
	The children are near the end of their path, but C11 hesitates when choosing the last arrow.	C11 holds up a forward arrow but pauses before placing it.	C11: “Will this get Kubo to the end?”	The teacher points to the end goal and nods.	“Let us think. If Kubo goes straight, will it reach the goal?”	C11 places the arrow and says, “Yes, it will reach the goal now.”
7	The children are confused about which arrow to place next to make Kubo turn around an obstacle.	C12 waves their hand over the tiles, unsure of which one to choose.	C12: “Should we turn here?”	The teacher points to the turn arrow and gestures toward the obstacle.	“Yes, what happens if we use this arrow?”	C12 places the turn arrow and says, “It will go around this way.”
	The children are stuck after making a mistake in the path.	C13 points to the section of the path where the mistake was made.	C13: “I think this part is wrong.”	The teacher traces the path with their finger, pointing to the mistake.	“Let us check each arrow. Where do you think the mistake happened? Was Kubo sleeping? I think so!”	C13 fixes the mistake and says, “Now it will work!”
8	The children are building a new path but are unsure how to start it.	C14 points to the starting arrow but hesitates to place it.	C14: “Should we start here?”	The teacher points to the start and nods.	“Yes, place the arrow here and let us see where Kubo goes.”	C14 places the first arrow and says, “Now it will go straight.”
	The children are revisiting an old path to improve their solution.	C15 moves their hand over the path, thinking about where to make changes.	C15: “What if we change this part?”	The teacher points to the section and nods in agreement.	“Yes, what happens if we use a different arrow here?”	C15 replaces the arrow and says, “It will go faster now.”
9	The children are approaching the final part of the path and need to make a sharp turn.	C16 hovers over the turning arrow but seems unsure about placing it.	C16: “Is this the right turn?”	The teacher points to the turning arrow and gestures toward the end goal.	“If Kubo turns here, where will it end up?”	C16 places the arrow confidently, saying, “It will go to the end!”
	The children are confused about which arrows to place after encountering a fork in the path.	C17 points back and forth between two arrows, unsure which one to choose.	C17: “Should we go left, or right?”	The teacher points to both options, encouraging the child to think.	“What happened last time when we made a choice like this?”	C17 remembers and says, “Last time we went left, so I’ll try left again.”

### 4.4 Comparison of Observations 0 and 1

The analysis of Observations 0 and 1 reveals significant differences in teachers’ mediation strategies and their impact on children’s communication during the problem-solving activities with the coding toy. In Observation 0, teachers predominantly relied on verbal instructions and often overlooked children’s non-verbal cues. For example, in Table 1, during an activity where C1 pointed to the left arrow tile and said, “Kubo should go there!,” the teacher responded by saying, “Let us move forward now,” without acknowledging the child’s gesture or suggestion. This led to decreased engagement from the child, who stopped pointing and simply agreed to move forward.

In contrast, Observation 1 illustrated a noticeable shift towards a multimodal mediation approach. Teachers incorporated both verbal and non-verbal communication, actively acknowledging and responding to children’s gestures. For instance, as shown in Table 2, when C1 pointed towards the robot’s path indicating his plan, the teacher acknowledged the gesture by nodding and asking, “Are you showing me where the robot should go next?” This encouraged the child to continue using gestures to communicate his strategy, facilitating his engagement and participation.

These differences highlight the importance of specific mediation elements identified during the workshop. Teachers’ use of gestures, open-ended questions, and providing time for children to think and respond contributed to a more inclusive and supportive learning environment. These strategies were effective across the 4.1 to 4.9 years age range, accommodating varying levels of verbal and motor skills among the children. In Observation 1, specific gestures such as pointing, tracing paths with fingers, and mirroring children’s actions played a crucial role in facilitating communication and participation in the problem-solving play-based activity. Teachers used gestures to draw attention to specific tiles or paths, helping children visualize the sequence of actions. For example, when C4 hesitated before placing the last arrow, the teacher traced the path with her finger, leading C4’s hand to visualize the final move, and said, “Take your time, C4, just think. What happens if you place that arrow? Where will Kubo go?” This non-verbal support, combined with open-ended questions, encouraged the child to think critically and make decisions independently. During Observation 1, teachers employed various types of feedback to support children’s communication and participation in the problem-solving processes. Affirming feedback, such as “Yes, that’s a good idea!,” validated children’s choices and supported their confidence. Directive feedback provided guidance when children were uncertain, for example, “If you place the turn here, where will Kubo go next?” Open-ended feedback encouraged children to reflect and think critically, as seen when the teacher asked, “What do you think will happen if we use this arrow?” These feedback strategies were effective in engaging children and promoting active participation.

## 5 Discussion

### 5.1 Mediation elements identified by the teachers are relevant to facilitate children’s communication

Coding toys like Kubo differ from traditional teaching materials in their ability to combine tactile interaction with logical sequencing, making abstract concepts more accessible to young children (Bertel et al., 2019). Unlike traditional manipulatives, coding toys engage children in iterative problem-solving processes that integrate technology into play-based learning. These unique features provide opportunities for fostering multimodal communication and critical thinking in ways that traditional tools may not.

The notes written by the teachers during the workshop presented several main themes related to the theory of semiotic mediation (Bartolini Bussi et al., 2011). The identified themes were the importance of planning, mediation elements during the activity, and a multimodal approach.

The teachers discussed the importance of planning the activities. Observation 0 was intended to observe teachers and children investigating a new artefact, the coding toy, together. Comments such as “We need to understand the problem in advance” and “We can investigate a new coding toy together with children, but then it is complicated to support their learning or understanding” illustrate teachers’ need to avoid improvisation, especially when supporting children’s exploration, understanding, and communication. This aligns with the theory of semiotic mediation, which identifies planning, choosing the artefact, and designing the task as important aspects of the process. With sufficient time for planning and a clearer understanding of the robot’s functionality, the teachers were able to focus more attentively on the children, becoming increasingly aware of the full range of their communication, including both verbal and non-verbal expressions. By intentionally planning activities that encourage inquiry and exploration, teachers could scaffold children’s engagement with problem-solving, promoting a gradual, structured discovery process that allows children to draw connections between tangible actions and abstract concepts (Bartolini Bussi et al., 2011). Teachers in the workshop aimed to support children’s understanding of the problem-solving task by encouraging them to explore Kubo’s functionality, interpret the sequence of actions, and recognize how each arrow functions as part of a larger plan. This approach enabled children to perceive the coding activity not merely as isolated actions but as a coherent mathematical structure requiring problem-solving and systematic thinking (Mariotti, 2013).

The multimodal approach of the teachers was central to the activities discussed during the workshop.

Teachers saw value in scaffolding children’s communication through a multimodal approach, combining verbal (“Teachers need to ask children to describe their actions”) and non-verbal support (“Teachers demonstrated the movements to help children”) to foster reflection (“What do you think will happen?” and “It is important to let the children think for themselves”) and collaborative problem-solving. According to Bartolini Bussi and Baccaglini-Frank (2015), teachers mediate children’s learning by bridging the gap between situated signs (like coding arrows) and broader mathematical signs, helping children internalize problem-solving strategies through a guided process. Teachers incorporated reflective questioning and non-verbal support to create what Vygotsky (1986) calls a zone of proximal development, where children can operate just beyond their independent ability with appropriate guidance. This multimodal approach helps children understand both the immediate task and its underlying mathematical principles, gradually transforming external actions into internalized knowledge (Bartolini Bussi and Mariotti, 2008).

The teachers reflected on the mediation approach, describing it as more than just a multimodal approach. In their mediation approach, other elements were mentioned, such as the importance of giving children time for understanding and answering and the possibility of using humour. In this study, teachers identified situated signs, such as the arrows and tiles, which serve as immediate, tangible representations of movement and direction. These signs allow children to manipulate and visualize Kubo’s path, a critical component in developing an understanding of spatial relations. McNeill (1992) and Roth (2001) argue that the physical manipulation of artefacts through gestures and positioning enables learners to engage with problem-solving on both an embodied and cognitive level. By engaging with situated signs, children actively participate in constructing meaning and transforming the arrows into semiotic representations that hold mathematical implications (Bartolini Bussi et al., 2011).

Finally, teachers in the workshop recognized the significance of the mathematical text inherent in Kubo’s coding activities. In Bartolini Bussi and Baccaglini-Frank’s (2015) theory, mathematical text refers to the formal mathematical ideas embedded within an artefact or task, which can emerge through guided interaction. In Kubo’s activities, the mathematical text involves concepts like orientation, sequence-building, and spatial awareness, essential for navigating the coding toy successfully and solving problem-solving tasks. Bartolini Bussi et al. (2011) highlight that the transition from artefact-specific understanding to abstract reasoning requires teachers to support children in connecting individual actions (like placing an arrow) to broader patterns and strategies (such as planning a path). Teachers’ efforts to link situated signs (arrows) with the overall path as a structured sequence align with this aspect of the theory. By framing coding actions as part of a coherent mathematical text, teachers helped children recognize and internalize the logical and sequential structures inherent in problem-solving activities, providing a bridge from concrete manipulation to abstract mathematical thought (Bartolini Bussi and Mariotti, 2008; Alibali and Nathan, 2012).

While this study focused on children aged 4.1 to 4.9 years, the mediational elements identified have implications for different age groups. Teachers noted that gestures and non-verbal cues were particularly supportive for children who might have limited verbal skills, while open-ended questions and reflective dialogue benefited children with more advanced language abilities. Recognizing the developmental differences, teachers adapted their strategies to meet the needs of individual children, highlighting the importance of flexibility in mediation approaches. These findings offer valuable insights for educators seeking to incorporate technology in a way that is engaging, developmentally appropriate, and pedagogically adapted. The study emphasizes the potential of coding toys to complement traditional teaching methods by integrating technology into play-based activities.

### 5.2 The importance of the mediation elements chosen in facilitating children’s communication

From the analysis of the observations conducted after the workshop (Observation 1), it is possible to affirm that the teachers applied the mediation elements they identified as relevant. Teachers used both verbal and non-verbal support. For example, it was observed that teachers used verbal mediation, such as “What do you think will happen if Kubo goes straight?” They also used non-verbal mediation, as in the example: “The teacher nods and points toward the forward arrow.” These strategies were observed more frequently in Observation 1 than in Observation 0. Moreover, in Observation 0, many gestures displayed by the children were not acknowledged. For instance, in the sequence: “Children and the teacher are sitting on the floor. They started to program, and C1 is trying to decide where the Kubo robot should go next. He points to the left arrow tile, signaling that Kubo should turn left. C1 says, ‘Kubo should go there!’ Then the teacher says, ‘Let us move forward now.’ C1 stops pointing and just says, ‘Okay, forward.’” This sequence highlights that while the teacher is listening to the child, the child’s multimodal communication is not taken into account.

A significant contrast is evident in the sequences observed in Observation 1. By focusing on multimodal communication, teachers enriched their interactions with children, fostering a communicative environment that facilitated both verbal and non-verbal expression. This development aligns with Bartolini Bussi and Baccaglini-Frank’s (2015) Theory of Semiotic Mediation, which emphasizes the role of teacher mediation in connecting artefact-based interactions to broader mathematical understanding. In accordance with this theory, situated signs, such as pointing to or manipulating coding tiles, serve as immediate cues that can guide children’s understanding within the activity’s physical context. During Observation 1, teachers used gestures, such as pointing to specific tiles or mimicking Kubo’s movements, to direct children’s attention and clarify the task’s requirements. This multimodal support allowed children to interpret and respond to cues beyond verbal instructions, bridging the gap between physical manipulation and abstract understanding (Goldin-Meadow, 2003). Children’s responses during Observation 1 reflected an increased reliance on gestures to communicate their ideas, an approach that further supported their verbal expression. Teachers’ consistent use of multimodal communication provided children with multiple channels for engagement, allowing them to express their thoughts much more frequently than in Observation 0. Studies show that combining verbal and non-verbal support in educational settings enhances cognitive processing by reinforcing multiple aspects of an idea simultaneously (Alibali and Nathan, 2012). This holistic approach encouraged children to explore the coding activity actively, using both speech and gestures to communicate and test their ideas.

The workshop’s focus on mediating strategies was clearly reflected in teachers’ interactions with children, as they applied strategies to break down complex tasks into smaller, manageable steps. Bartolini Bussi and Baccaglini-Frank’s framework suggests that teacher mediation should help children transition from understanding artefact-based actions to engaging with abstract concepts, a process achieved through carefully structured support (Bartolini Bussi et al., 2011). During Observation 1, teachers implemented scaffolding techniques by encouraging children to identify, analyze, and experiment with individual steps in the coding sequence, such as placing each arrow in sequence to build Kubo’s path. The observations revealed that children responded positively to this scaffolding, often repeating the steps and expressing their reasoning. Teachers’ questions, such as “What do you think will happen next?” or “How does this arrow help Kubo reach the goal?,” encouraged reflective thinking and verbalization of the problem-solving process. This aligns with Vygotsky’s (1986) theory that guided interactions within a zone of proximal development enable children to engage in tasks beyond their independent capacity. By breaking down the task, teachers helped children gain confidence in solving parts of the problem, eventually connecting these steps to achieve the overall objective.

The varied feedback provided by teachers played a significant role in supporting children’s motivation during the activities. Affirming feedback reinforced positive actions and decisions, increasing children’s confidence in their abilities. Open-ended questions stimulated curiosity and encouraged deeper thinking, leading to greater engagement. For instance, when teachers allowed children time to think and provided hints through gestures or questions, children were more likely to persist in problem-solving tasks and express satisfaction upon finding solutions.

Among the mediation strategies discussed during the workshop, two are particularly noteworthy. One is giving children time to think and answer, as seen in the sequence: “The children have almost completed their path but need one final arrow to reach the goal. C4 points to the endpoint but hesitates before placing the last arrow. ‘Will this make Kubo reach the end?’ The teacher waits and gives C4 time to think. She sees that C4 tries to trace the path with their finger and then traces the path with her finger, leading C4’s hand to visualize the final move. ‘Take your time, C4, just think. What happens if you place that arrow? Where will Kubo go?’ C4 places the arrow and says, ‘It will reach the end now.’” This sequence demonstrates that the child was given the opportunity to participate and suggest a solution even if they were not completely confident.

The second element is humor, as illustrated in the sequence: “The children are stuck after making a mistake in the path. C13 points to the section of the path where the mistake was made. C13: ‘I think this part is wrong.’ The teacher traces the path with their finger, pointing to the mistake. ‘Let us check each arrow. Where do you think the mistake happened? Was Kubo sleeping? I think so!’ C13 fixes the mistake and says, ‘Now it will work!’” Research shows that humor helps children reduce stress, makes the activity feel less challenging, and encourages inclusive participation (Bishara, 2023; Gazit, 2018).

While our findings align with the principles highlighted in Bartolini Bussi and Baccaglini-Frank’s (2015) study, it is important to note the differing scopes and methodologies. Unlike their research, which was conducted with a homogeneous group over a prolonged period with direct mathematical interventions, our study focuses on teacher mediation strategies within the Norwegian ECEC context. This study complements Bartolini Bussi and Baccaglini-Frank’s work by exploring how multimodal mediation supports children’s communication during problem-solving activities with coding toys, without attempting to directly confirm their results.

## 6 Implications and suggestions

The findings from this study underscore the critical role of teacher mediation in facilitating children’s communication during a problem-solving play-based activity with coding toys like Kubo. By incorporating multimodal communication, reflective questioning, and intentional planning, teachers can create rich, supportive environments that foster both verbal and non-verbal expression among children. This study suggests several practical implications for ECEC practitioners and provides directions for further development in early childhood education. It is important to note that the scope of this study is limited to examining teachers’ mediation strategies rather than assessing children’s problem-solving competence. While teachers’ facilitation may indirectly support skill development, these findings are not presented here and should be further explored in future research.

The integration of mediation through multimodal support, such as gestures, verbal scaffolding, and reflective questioning, should be emphasized in teacher training programs. These techniques encourage children to articulate their thoughts and collaborate with peers, making communication a central part of problem-solving activities. Providing ECEC teachers with training on interpreting and using children’s non-verbal expressions, such as gestures, alongside verbal scaffolding, can enrich children’s learning experiences and help them build confidence in expressing ideas.

Findings highlight the importance of teachers’ planning when introducing coding toys or similar artefacts in ECEC settings. Planning activities that include both exploratory and goal-oriented elements enables teachers to provide a balance of structure and flexibility, adapting to the needs of different learners.

The findings suggest that periodic collaborative sessions, in addition to a single workshop, could enhance teachers’ ability to refine and adapt their mediation strategies. Regular opportunities for shared reflection could allow teachers to compare experiences, deepen their understanding of mediation techniques, and track the evolution of their practices over time.

Future research should explore how mediational strategies impact children across different age groups. Comparative studies could examine the effectiveness of specific mediation elements for younger versus older children, providing insights into tailoring approaches based on developmental stages. This would enhance the applicability of findings and support teachers in implementing age-appropriate strategies to facilitate communication and participation in problem-solving processes.

## 7 Limitations

While this study provides insights into the role of teacher mediation in facilitating children’s communication during a problem-solving play-based activity with a coding toy, it has several limitations. First, the study was conducted within a limited number of ECEC institutions, which may restrict the generalizability of the findings to other educational settings. Additionally, while the use of video recordings allowed for in-depth analysis of teacher-child interactions, the presence of recording equipment may have influenced the natural behaviors of both teachers and children, potentially introducing observational bias. The study also focused primarily on the initial stages of problem-solving, particularly understanding the problem; further research is needed to examine how mediation strategies may influence the later stages of problem-solving. Lastly, although the total duration of this study was 6 months (starting with Observation 0, followed by regular weekly activities, the workshop, continued weekly activities, and Observation 1), the teachers collaborated directly only during the workshop, where they compared their experiences and reflections. Although they had the opportunity to interact continuously through the online platform, it was observed that the platform was rarely used for this purpose. This means that after the single day of direct collaboration, the teachers worked individually based on the suggestions shared during the workshop. A more regular schedule for collaboration, such as biweekly or monthly meetings, could have been beneficial for observing how these elements evolved during their application. Post-Observation 1 reflections were conducted individually by the teachers in collaboration with the first author. Subsequent workshops, as part of the larger project, did not specifically focus on communication. Future studies could extend the duration and diversity of training to provide a more comprehensive understanding of how teachers can effectively integrate multimodal approaches to foster children’s communication and support their learning in coding activities.

Another limitation of this study is its focus on a narrow age range of children aged 4.1 to 4.9 years, which may limit the generalizability of the findings to other age groups. Additionally, the six-month gap between Observation 0 and Observation 1 may have introduced developmental and cognitive changes in the children that were not captured in the teachers’ logs. While weekly activities with the Kubo robot provided some consistency, the lack of a standardized curriculum, researchers’ observations during this period, and the limited use of the online platform for collaborative reflection may have influenced the findings. Future studies could include more frequent collaborative sessions to better monitor the evolution of mediational strategies over time.

Furthermore, this study did not include comparisons with other educational methods or tools, which could provide a broader perspective on children’s communication and exploration of problem-solving situations in ECEC settings. While this study presents the potential of coding toys to complement traditional teaching methods by integrating technology into play-based activities, future research could explore comparative studies to determine how coding toys and traditional materials uniquely support different aspects of children’s cognitive and communicative development.

## 8 Conclusion

This study investigates the mediation elements that teachers can identify as relevant to support children’s communication (verbal and non-verbal) during problem-solving play-based activities with a coding toy. Through the lens of Bartolini Bussi and Baccaglini-Frank’s (2015) Theory of Semiotic Mediation, we analyzed how key elements identified during a teacher workshop, such as multimodal communication, scaffolding, and reflective problem-solving, supported teachers’ ability to consider both verbal and non-verbal interactions. Our study builds on Bartolini Bussi and Baccaglini-Frank’s theoretical framework, adapting it to the Norwegian ECEC context. While their findings were derived from a structured educational experiment, our study offers a complementary perspective by examining teacher mediation strategies in play-based problem-solving activities. These findings contribute to understanding how theoretical principles can inform practice in diverse educational contexts. The data collected during this study, particularly the comparison between the results of Observation 1 and those of Observation 0, suggest that careful preparation of activities and a multimodal mediation approach enable teachers to pay greater attention to the communication used by children and to facilitate it effectively, especially in the context of a play-based problem-solving activity with a coding toy. These findings support the literature showing the value of integrating coding toys in ECEC settings and the importance of training teachers to plan activities and apply multimodal, scaffolded approaches that support children’s holistic development. By applying Bartolini Bussi and Baccaglini-Frank’s Theory of Semiotic Mediation in a practical context, this study investigates how teachers can bridge concrete experiences with abstract concepts through purposeful mediation. The use of physical artefacts, such as Kubo’s tiles, combined with multimodal communication strategies, enables children to construct mathematical understanding in a play-based environment. This translation of theory into practice highlights the critical role of teachers in facilitating learning through semiotic resources.

## Data Availability

The datasets presented in this article are not readily available because are video recordings where children were playing. Anonymised transcriptions can be available after request. Requests to access the datasets should be directed to Francesca Granone, francesca.granone@uis.no.
